# Hardness, Cohesiveness, and Adhesiveness of Oral Moisturizers and Denture Adhesives: Selection Criteria for Denture Wearers

**DOI:** 10.3390/dj4040034

**Published:** 2016-10-03

**Authors:** Keiko Fujimoto, Norikazu Minami, Takaharu Goto, Yuichi Ishida, Megumi Watanabe, Kan Nagao, Tetsuo Ichikawa

**Affiliations:** Department of Oral & Maxillofacial Prosthodontics, Tokushima University, Graduate School of Biomedical Sciences, 3-18-15, Kuramoto, Tokushima 770-8504, Japan; c301451014@tokushima-u.ac.jp (K.F.); minamino@tokushima-u.ac.jp (N.M.); tak510@tokushima-u.ac.jp (T.G.); megwat@tokushima-u.ac.jp (M.W.); kan@tokushima-u.ac.jp (K.N.); ichi@tokushima-u.ac.jp (T.I.)

**Keywords:** denture adhesives, oral moisturize, texture profile analysis, hardness, cohesiveness, adhesiveness

## Abstract

The mechanical properties of seven denture adhesives and eight oral moisturizers, all of which are commercially available, were evaluated using a texture profile analysis. A new assessment chart is proposed for the selection criteria of denture adhesive and oral moisturizers using a radar chart with three axes: hardness, cohesiveness, and adhesiveness.

## 1. Introduction

The number of denture wearers who have dry mouth and difficulty chewing has been increasing in ultra-aged society and particularly in Japan. Dry mouth is extremely common in the elderly who regularly administer prescription or nonprescription medication for radiation therapy, diabetes mellitus, and Shögren syndrome [1,2,3,4]. An oral moisturizer, often called “artificial saliva,” is used as a symptomatic treatment for dry mouth. Dry mouth in denture wearers often results in denture instability and denture complications despite the dentures being of the appropriate form and fitness. In this case, oral moisturizers improve the denture problems due to dry mouth [5,6]. Severe bone resorption among the elderly, which occurs due to extension of life, may also result in denture instability. Even the most accomplished dentists find it difficult to satisfy patients’ expectations for stability and retention of the denture, and it is occasionally considered appropriate to prescribe a denture adhesive to the patients [7,8,9,10]. Although various denture adhesives and oral moisturizers are commercially available, no product possesses all the required characteristics of the materials, namely adhesiveness, moisture, flow and thickness, and ease of removal, among others. Patients often hesitate (or are unable) to select the appropriate material for their specific situation, and may need to be guided via a case-specific selection criteria.

In this study, texture profile analysis (TPA), which is a popular double-compression test for determining the textural properties of food material [11,12], was used to examine the material properties of commercial denture adhesive and oral moisturizers. The selection criteria for denture adhesives and oral moisturizers are discussed based on the TPA. 

## 2. Material and Methods

### 2.1. Tested Materials

Table 1 shows the description of eight oral moistures and seven denture adhesives that were evaluated in this experiment. All materials are commercially available in Japan and throughout the world.

### 2.2. Measurements of Material Properties

Seven oral moisturizers and eight denture adhesives were tested using TPA, which is a popular double compression test for food material [11,12,13,14].

**Figure 1:**
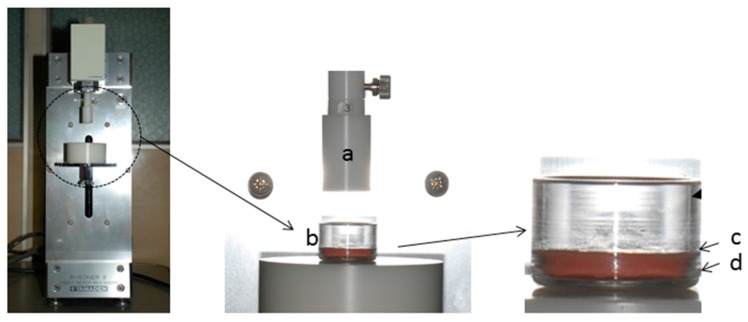
Creep meter (**Left**) and its compressive condition (**Center**, **Right**). (**a**) Plunger; (**b**) Glass dish; (**c**) Type 1 collagen cell desk; (**d**) Silicone impression material.

**Figure 2:**
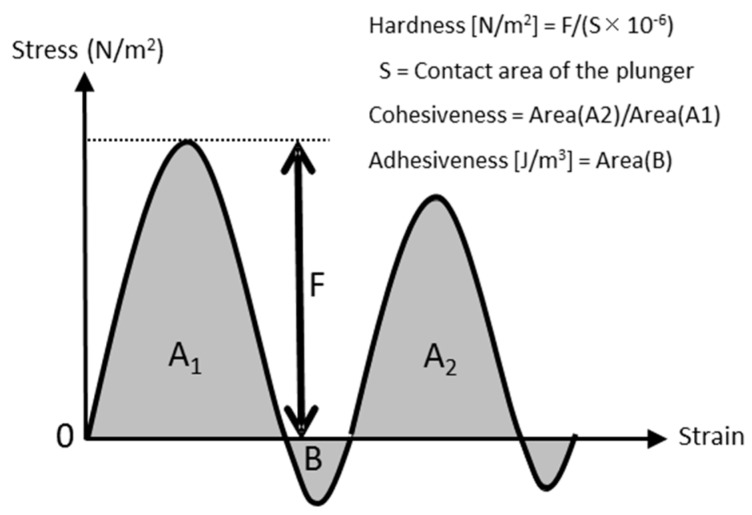
Texture analysis for hardness, cohesiveness, and adhesiveness of materials.

Figure 1 and Figure 2 show the measurement system and analysis method. A type I collagen-coated plastic plate (Celldesk LF1, MS-92132, SUMITOMO BAKELITE, Tokyo, Japan) lined with a 3-mm-thick silicone impression material (EXAFINE Injection-type, GC, Tokyo, Japan) (adopted as a simulated mucosa [15,16]) was placed on the bottom of an 18-mm-diameter glass dish. A 0.2-g weight material was directly placed on the simulated mucosa as homogeneously as possible. The materials were evaluated according to a TPA formula, based on the stress–strain curve obtained using a creep meter (RE2-3305B, Yamaden, Tokyo, Japan) with a flat piston head (ø of 16 mm), cylindrical glass dish, and a 2-N load cell. The creep meter measures the tendency of a solid material to move slowly or deform permanently under the mechanical stresses. Each material underwent two successive compression cycles performed at a constant displacement rate of 1 mm/s. The material was compressed 50% of the original height, and the return height at the secondary compression was 5 mm above the original height. In this analysis, hardness was defined as the maximal stress (force divided by the bottom area of the plunger) reached during the first compression. Adhesiveness was calculated using the area over the negative stress–strain curve after the first compression, which represents the work per unit volume. Cohesiveness was defined as the ratio of the area under the second compression curve to the area under the first compression. Measurements of each material were repeated ten times, and the means were considered as the representative values.

## 3. Results

**Figure 3:**
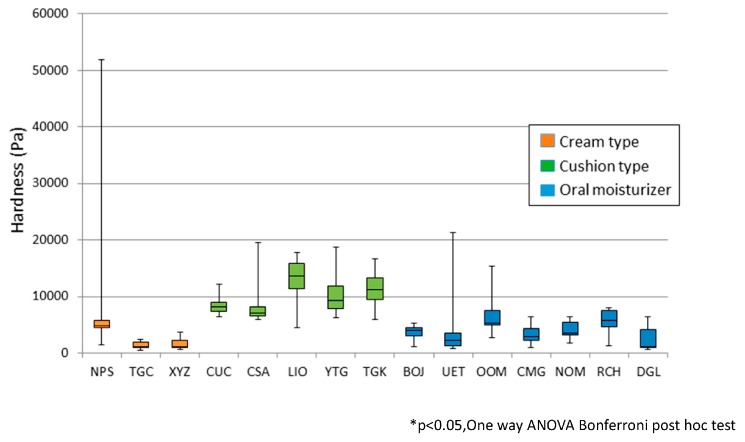
Hardness of seven kinds of denture adhesives and eight kinds of oral moisturizers.

**Figure 4:**
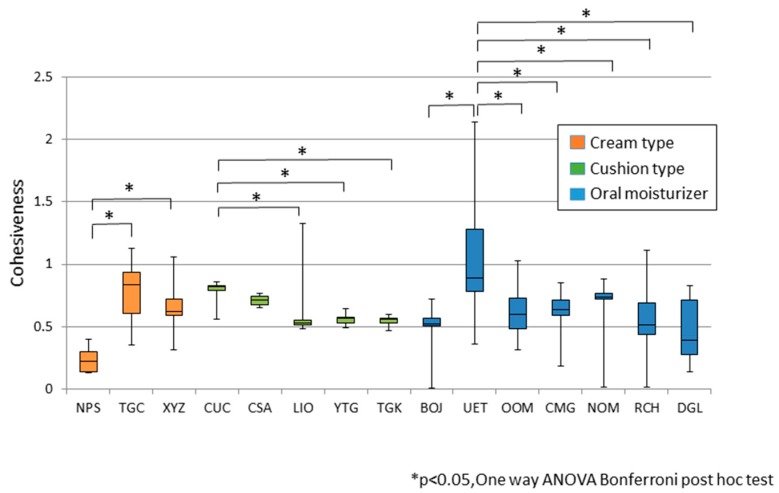
Cohesiveness of seven kinds of denture adhesives and eight kinds of oral moisturizers of seven kinds of denture adhesives and eight kinds of oral moisturizers.

**Figure 5:**
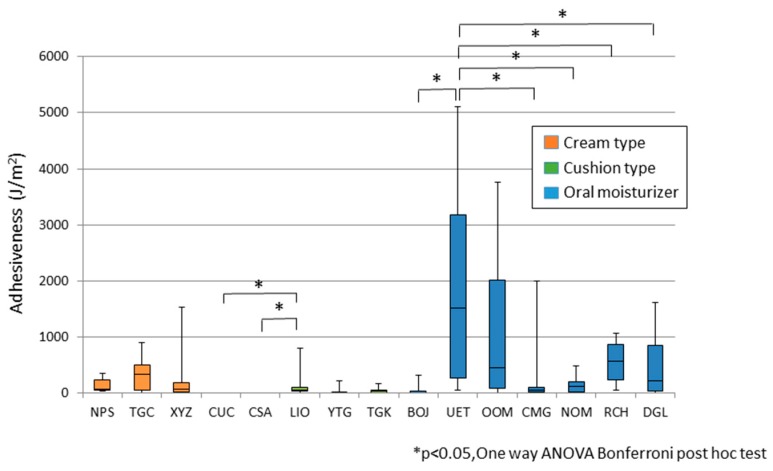
Adhesiveness of seven kinds of denture adhesives and eight kinds of oral moisturizers.

Figure 3 shows the hardness of each tested material, categorized into three groups. The bottom and top of the box, the ends of the whiskers, and the band inside the box represent the lower (first) quartile, upper (third) quartile, minimum, maximum, and median, respectively (the same is with Figure 4 and Figure 5). The hardness of the cushion-type denture adhesives was higher than that of the cream-type denture adhesives and oral moisturizers. The hardness of NPS varied widely. The NPS was brittle with a powdery feeling and might have influenced the measurement.

Figure 4 shows the cohesiveness of each tested material, categorized into three groups. The cohesiveness was characterized not by group, but on an individual material basis. 

Figure 5 shows the adhesiveness of each tested material, categorized into three groups. The adhesiveness of the oral moisturizers was comparably higher than that of the denture adhesives.

## 4. Discussion

Denture wearers are often not sure of which denture adhesive or oral moisturizer is appropriate for them. Patients clarify their oral or denture complications and then select the appropriate material for addressing the complications. While clarification regarding the material and biological characteristics of the materials is required prior to the selection of the material, it is very difficult for dentists and patients to understand the material characteristics and how they relate to findings from rheological research.

Oral moisturizers are formulated to improve dry mouth. Denture wearers with dry mouth need a lubricant agent as well as a moisturizer as a substitute for saliva. Commercial oral moisturizers consist of moisturizing agents, supplements, and water. Glycerin is generally used as the moisturizing agent, in conjunction with the hyaluronic acid. Sweetener, essence, and antibacterial agents, such as lactoferrin, lactoperoxidase, and lysozyme, are generally compounded as supplements [5,6]. 

The denture adhesive requires retention and stability without altering the interocclusal relationship. The additional requisite is the removal of the material from the denture base. The denture adhesives are categorized into cream type, cushion type (home reliner type), powder type, and tape type. The principal component of powder type and cream type denture adhesives is carboxymethyl cellulose sodium salt, and the principal components of cushion type adhesives are polyvinyl acetate and ethanol [17]. Any denture adhesive and oral moisturizer has little biological hazard, a good feeling of prescription, and an antibacterial effect.

In this study, TPA with three texture parameters was applied to evaluate the mechanical properties of denture adhesives and oral moisturizers. This method is generally used not only for food analysis, but also for analyses of pharmaceuticals, gels, and items of personal care. In TPA, test samples are compressed twice using a texture analyzer to evaluate the behavior of samples when chewed. The method recognizes the fact that the textural identity of any material is both multi-faceted and inherently linked to the patient's sensory expectations. The hardness refers to a deformationability that spreads between denture and oral mucosa, whereas cohesiveness refers to the retention capacity of the material, which is the ratio of total energy at first compression to total energy at second compression. The high hardness secures the material thickness for the inadaptation of denture, and high cohesiveness shows that the material can be uniformly coated to the denture. The adhesiveness refers to the energy required to separate two materials and represents the extent of denture retention. 

**Figure 6:**
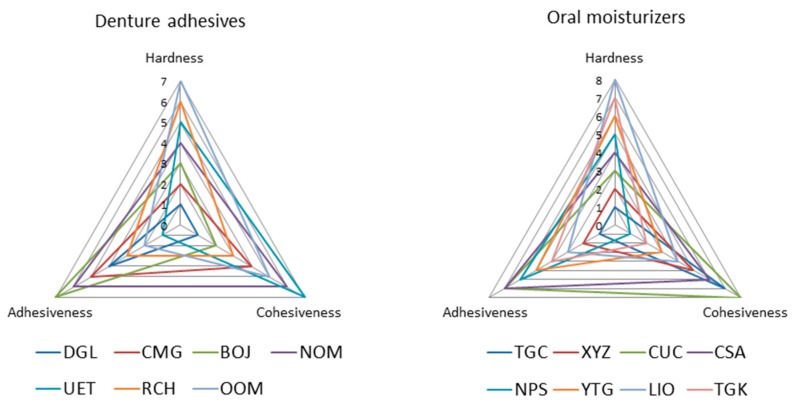
Radar charts with three axes (**Left**: denture adhesives; **Right**: oral moisturizers).

A radar chart with three axes (i.e., hardness, cohesiveness, and adhesiveness) makes it easier to comprehend the characteristic of the material, as shown in Figure 6. The radar chart is available for seeing which properties are scoring high or low within a dataset, making them ideal for displaying performance. If the patient needs enhanced denture retention until the adaptation to a new denture, the denture adhesive or oral moisturizer with low hardness and low adhesiveness will be preferable. If the patient needs enhanced denture retention for a long term, a denture adhesive or oral moisturizer with high hardness and high cohesiveness may be preferable. The radar chart will be helpful in selecting the appropriate oral moisturizer and denture adhesive. The relationship between the main component and radar chart outline of each material was not found, and every material showed individual original features. 

In conclusion, our study illustrated a new assessment chart for the selection criteria of denture adhesive and oral moisturizers using a radar chart with three axes: hardness, cohesiveness, and adhesiveness through texture profile analysis. This will provide both patients and dentists with information useful for denture maintenance.

## Figures and Tables

**Table 1:** Tested materials.

	Commercial Name	Products	Code
Oral Moisturizers	Biotene Oralbalance jel^®^	GlaxoSmithKline (Tokyo, Japan) *	BOJ
	Wet-aid	Kamemizu Chem. Ind (Osaka, Japan)*	UET
	Optreoz^TM^ oral Moisturizer	3M Japan (Tokyo, Japan) *	OOM
	ConCool Mouth Gel	Weltec (Osaka, Japan) *	CMG
	New Oral Moisturizer Ai Gel	Hishika Dental (Mie, Japan)	NOM
	Rifre-care^®^ H	EN Otsuka Pharmaceutical (Iwate, Japan) *	RCH
	Denture Gel	Kamemizu Chemical Ind (Osaka, Japan) *	DGL
Denture Adhesives	Cream-type		
	New Poligrip^®^ Sa	GlaxoSmithKline (Tokyo, Japan) *	NPS
	Tough grip^®^ cream	Kobayashi Pharmaceutical (Osaka, Japan) *	TGC
	Correct^®^ XYL Cream	Shionogi Healthcare (Osaka, Japan) *	XYZ
	Cushion-type		
	Cushion Correct^®^	Shionogi Healthcare (Osaka, Japan) *	CUC
	Correct^®^ Soft A	Shionogi Healthcare (Osaka, Japan)	CSA
	Liodent	Lion (Tokyo, Japan) *	LIO
	Yawaraka Tafugurippu^®^	Kobayashi Pharmaceutical (Osaka, Japan) *	YTG
	Tafugurippu^®^ Kusshon	Kobayashi Pharmaceutical (Osaka,Japan) *	TGK

* Product is available worldwide through mail order or some way.

## References

[1] Diaz-Arnold A.M., Marek C.A. (2002). The impact of saliva on patient care: A literature review. J. Prosthet. Dent..

[2] Ikebe K., Morii K., Kashiwagi J., Nokubi T., Ettinger R.L. (2005). Impact of dry mouth on oral symptoms and function in removable denture wearers in Japan. Oral Surg. Oral Med. Oral Pathol. Oral Radiol. Endod..

[3] Ikebe K., Amemiya M., Morii K., Matsuda K., Furuya-Yoshinaka M., Yoshinaka M., Nokubi T. (2007). Association between oral stereognostic ability and masticatory performance in aged complete denture wearers. Int. J. Prosthodont..

[4] Tanasiewicz M., Hildebrandt T., Obersztyn I. (2016). Xerostomia of Various Etiologies: A Review of the Literature. Adv. Clin. Exp. Med..

[5] Gil-Montoya J.A., Guardia-López I., González-Moles M.A. (2008). Evaluation of the clinical efficacy of a mouthwash and oral gel containing the antimicrobial proteins lactoperoxidase, lysozyme and lactoferrin in elderly patients with dry mouth—A pilot study. Gerodontology.

[6] Murakami M., Nishi Y., Fujishima K., Nishio M., Minemoto Y., Kanie T., Nishimura M. (2015). Impact of Types of Moisturizer and Humidity on the Residual Weight and Viscosity of Liquid and Gel Oral Moisturizers. J. Prosthodont..

[7] Papadiochou S., Emmanouil I., Papadiochos I. (2015). Denture adhesives: A systematic review. J. Prosthet. Dent..

[8] Kumar P.R., Shajahan P.A., Mathew J., Koruthu A., Aravind P., Ahammed M.F. (2015). Denture Adhesives in Prosthodontics: An Overview. J. Int. Oral Health.

[9] Felton D., Cooper L., Duqum I., Minsley G., Guckes A., Haug S., Meredith P., Solie C., Avery D., Deal Chandler N. (2011). Evidence-based guidelines for the care and maintenance of complete dentures: A publication of the American College of Prosthodontists. J. Prosthodont..

[10] Felton D., Cooper L., Duqum I., Minsley G., Guckes A., Haug S., Meredith P., Solie C., Avery D., Chandler N.D. (2011). Evidence-based guidelines for the care and maintenance of complete dentures: A publication of the American College of Prosthodontists. J. Am. Dent. Assoc..

[11] Bourne M.C. (2002). Food Texture and Viscosity: Concept and Measurement.

[12] Bourne M.C. (2004). Relation between texture and mastication. J. Texture Stud..

[13] Goto T., Nakamich A., Watanabe M., Nagao K., Matsuyama M., Ichikawa T. (2015). Influence of food volume per mouthful on chewing and bolus properties. Physiol. Behav..

[14] Wada S., Goto T., Fujimoto K., Watanabe M., Nagao K., Ichikawa T. (2016). Changes in food bolus texture during. J. Texture Stud..

[15] Ohguri T., Kawano F., Ichikawa T., Matsumoto N. (1999). Influence of occlusal scheme on the pressure distribution under a complete denture. Int. J. Prosthodont..

[16] Emmer T.J., Emmer T.J., Vaidyanathan J., Vaidyanathan T.K. (1999). Measurement of submucosal forces transmitted to dental implants. J. Oral Implantol..

[17] Kano H., Kurogi T., Shimizu T., Nishimura M., Murata H. (2012). Viscosity and adhesion strength of cream-type denture adhesives and mouth moisturizers. Dent. Mater. J..

